# A Live Birth Subsequent to IVF following Egg Retrieval Only 12 Hours after hCG Priming

**DOI:** 10.1155/2013/634385

**Published:** 2013-05-16

**Authors:** Limor Man, Joel Baron, Iris Har-Vardi, Eitan Lunenfeld, Eliahu Levitas

**Affiliations:** Fertility and IVF Unit, Department of Obstetrics & Gynecology, Soroka University Medical Center, Faculty of Health Sciences, Ben-Gurion University of the Negev, P.O. Box 151, Beersheva 84101, Israel

## Abstract

*Introduction*. To report a live birth following egg retrieval after only 12 hours from hCG priming. *Patients*. A childless couple with five-years-lasting secondary infertility. *Methods*. IVF was performed according to the long protocol. Two immature oocytes were retrieved following only 12 hours after hCG priming due to the patient misunderstanding. The eggs were cultured *in vitro* and ICSI was performed following polar body extruded after 24 hours in culture. After additional 24 hours a 4-cell embryo was developed and ET was performed. *Results*. A viable pregnancy was achieved and a healthy baby girl was delivered at 38 weeks of gestation. *Conclusion*. In a rare and unexpected situation when immature oocytes are retrieved following a short hCG priming, the eggs should be cultured *in vitro,* late ICSI should be performed, and a pregnancy may be expected.

## 1. Introduction

The luteinizing hormone (LH) surge stimulates a collection of events and ultimately leads to ovulation, the physical release of the oocyte, and its cumulus mass of granulose cells [1]. The LH surge initiates the continuation of meiosis in the oocyte, luteinization of granulose cells, expansion of the cumulus, and synthesis of prostaglandins essential for follicle rupture. The onset of the LH surge is the most reliable indicator of impeding ovulation, occurring 34–36 hours prior to follicle rupture [2]. A threshold of LH concentration must be maintained for at least 14–27 hours in order for full maturation of the oocyte to occur [3]; usually the LH surge lasts 48–50 hours [2].

During controlled ovarian hyperstimulation (COH), the natural endogenous LH surge usually does not appear. The interval between human chorionic gonadotropin (hCG) priming and oocyte retrieval is very important, because a series of crucial processes, such as the start of luteinization, expansion of cumulus cells, and the resumption of oocyte meiosis, is accomplished in that interval [4].

Like the LH surge in natural cycles, hCG triggers the resumption of meiosis in primary oocytes previously arrested at prophase I of the first meiotic division. hCG is used for final oocyte maturation. Nader and Berkowitz [5] studied the pharmacokinetics of hCG and its relation to ovulation and concluded that ovulation may occur 36 hours earlier in some women; they advised aiming for a <35-hour interval if ovulation is to be avoided. In most *in vitro* fertilization (IVF) programs, the commonly practiced interval is from 32 to 36 hours, which was derived from the studies on patients who used clomiphene citrate and human menopausal gonadotropin (hMG) for ovulation induction [6, 7]. Several studies [8, 9] have shown that ideal ART performance can be obtained when oocyte retrieval was done more than 36 hours (up to 39 hours) after hCG priming. The prolonged luteinization-to-oocyte retrieval increased the production of oocytes with fully expanded cumulus, which may reflect oocyte maturation; the researchers presumed that the subsequent proportion of oocytes proceeding to fertilization and cleavage also increased, implying that a longer interval may improve gamete quality by allowing more optimal *in vivo* maturation. Significantly more high-quality cleaving embryos were reported to be obtained when the interval was 38 hours rather than 36 hours [8]. On the other hand, other studies [10, 11] indicated that there was no significant difference on the outcome of IVF treatment cycles among the interval-prolonged cycles. A meta-analysis was carried out by Wang et al. [12] in order to determine whether a prolonged hCG-to-oocyte retrieval interval is beneficial to ART outcome. They demonstrated that the oocyte maturation rate was higher in the long-interval group compared with the short-interval group. They concluded that the percentage of mature (MII) oocytes can be increased by prolonging the interval between hCG priming and oocyte retrieval.

Although the large majority of immature oocytes will mature with time in culture (up to 36 hours) and may be fertilized, the embryos they generate often develop poorly and yield relatively poor pregnancy rates [13, 14].

We present a rare situation where due to a misunderstanding oocytes were retrieved only 12 h following hCG injection, and nevertheless a viable pregnancy was achieved.

## 2. Case Report

A childless couple with secondary infertility married for 5 years was admitted to our IVF Unit in 2010, due to prolonged anovulatory infertility and failure to conceive with repeated ovulatory cycles of gonadotropins and IUI.

The 27-year-old patient had irregular menses since menarche at the age of 14 years. Her past medical history was unremarkable and her BMI was 20. Physical examination did not reveal acne or hirsutism. Pelvic sonography showed normal pelvic findings with regular endometrium and polycystic ovaries. The early follicular phase FSH was 5.6 mIu/mL, LH was 9.4 mIu/mL, and prolactin and thyroid function levels were in the normal range. Hysterosalpingography demonstrated a normal shape uterine cavity and bilateral patent fallopian tubes. Obstetric history: two previous spontaneous pregnancies that ended in first-trimester spontaneous abortions.

The 36-year-old husband had diabetes mellitus type 2 and dyslipidemia and was a smoker. Andrological physical examination revealed normal findings. Semen analysis demonstrated isolated teratozoospermia 0-1% normal cells, its volume was 1.3–1.8 mL, and its concentration was 52–96 × 10^6^/mL with 74-75% motility.

During this first oocyte retrieval cycle, controlled ovarian hyperstimulation (COH) was achieved using pituitary suppression with daily injections of GnRH analog triptorelin (Decapeptyl: Ipsen, Paris, France) 0.1 mg starting at the preceding midluteal phase, and ovarian stimulation was commenced using recombinant follitropin alfa (Gonal F: Merck Serono Pharmaceuticals, Switzerland) at a dose of 75 U for 12 days. Three days before egg collection the patient had demonstrated on ultrasound ten follicles of 15–24 mm and estradiol level of 759 pg/mL. The patient was instructed to inject 75 U of follitropin alfa for one more day and 250 mcg of choriogonadotropin alfa (Ovitrelle: Merck Serono Pharmaceuticals, Switzerland) 36 hours prior to the egg retrieval. Only two oocytes were retrieved transvaginally. The oocytes were at first meiosis (M1 phase).

On review of the patient's instruction chart it was realized that she had injected the hCG 12 and not 36 hours prior to the egg collection as instructed. Following 24 h culture in a regular IVF medium (Sage media CooperSurgical), both eggs had extruded a polar body and undergone ICSI. Forty-eight hours from egg retrieval one oocyte showed a 2 PN state and the other showed 3 PN. Following an additional 24 h, namely, 72 hours from egg collection and 48 hours from the ICSI, the 2 PN zygote had developed into a 4-cell embryo, with good embryo morphology, and was transferred into the uterine cavity under transabdominal sonography. The luteal phase support included 300 mg/day of vaginal progesterone (Endometrin, Ferring Pharmaceuticals) and 4 mg/day of ethinyl estradiol (Estrofem, Novo Nordisk). Two weeks later the *β*hCG level was 376 mIU/L, progesterone was 95 ng/mL, and estradiol was 797 pg/mL. Transvaginal ultrasound examination two weeks later revealed a single intrauterine pregnancy and fetal heart beat. She delivered a healthy baby girl at 38 weeks of gestation, weighing 2930 grams, by cesarean section due to breech presentation.

## 3. Discussion

To the best of our knowledge this is the first publication of a viable pregnancy following egg retrieval only 12 h after hCG injection. The interval between hCG priming and oocyte aspiration is of paramount importance because of a series of crucial processes at the level of oocyte and cumulus cells.

Human oocytes are arrested in prophase I of meiosis (germinal vesicle) during fetal life. Following LH surge, the nuclear membrane dissolves, germinal vesicle (GV) is broken down, and the chromosomes progress from metaphase I to telophase I. The completion of the first meiotic division is characterized by expulsion of the first polar body and formation of the secondary oocyte containing a haploid set of chromosomes. The second meiotic division is initiated rapidly after the completion of the first meiotic division, and the oocytes reach metaphase II prior to ovulation. Oocyte maturation is defined as the transition from the germinal vesicle stage to metaphase II, accompanied by cytoplasmic maturation necessary for fertilization. Oocyte maturation combines complicated intra- and extracellular processes. The oocyte and cumulus oophorus cells are coupled by gap junctions, which allow control of the GV breakdown via the cumulus cells. The gap junctions permit regulatory molecules, such as steroids, Ca^2+^, inositol 1,4,5-trisphosphate (IP3), cAMP, and purines, to pass freely between the cytoplasm of the oocyte and cumulus cells [15]. The nuclear maturation is initiated by either luteinizing hormone (LH) surge or the atretic degeneration of the follicle. The competence to undergo GVBD was previously thought to be acquired during follicle growth. GV breakdown is a multistep process. In humans, usually some germinal vesicle oocytes collected from both stimulated and unstimulated ovaries fail to undergo GVBD during culture *in vitro*. It seems that the competence to undergo GVBD is only acquired during follicle growth, suggesting that an inherent capability for GVBD is still important.

Cumulus cells respond to gonadotropins and support the control of the nuclear and cytoplasmic maturation.

It is well accepted [16] that cycles using GnRHa COH cause LH suppression and therefore prolonged *in vivo* oocyte maturation time compared to cycles without the use of GnRHa. Immature oocytes are firmly attached to the follicular wall and most of the time impossible to aspirate using IVF and not IVM technique [17]. Van Steirteghem et al. [18] reported that when oocytes were retrieved 33–36 h after hCG priming, 85% were in the M2 state. Jagiello et al. [19] found that most oocytes were in M1 although some were in M2 by 28 h following hCG administration. In a natural human cycle, ovulation occurs between 40 and 63 hours after the E2 peak in the peripheral serum, indicating ovulation 16–39 h following LH surge [20]. The continuously acting GnRHa combined treatment suppresses the LH level and probably reduces the variation in the response to hCG.

In this particular case no IVM technique was applied and the oocytes were aspirated using a regular 17 G IVF needle as well as the laboratory technique of egg retrieval from the follicular fluid. The culture medium was the one we are using for regular IVF and did not contain FSH or LH.

The oocytes were fertilized successfully after 24 h in culture using late ICSI relative to oocyte collection. A live birth was achieved despite the observation of Tsirigotis et al. [21] that late ICSI (24 hours) can result in good fertilization and cleavage rates; however, the potential of the generated embryos to achieve pregnancy seems to be low. Using IVM, the implantation rates, clinical pregnancy rates, and live birth rates are lower than those in regular IVF [22].

We may conclude that in rare and unexpected situations when immature oocytes are aspirated following a short hCG priming, they should be cultured *in vitro*, late ICSI should be performed, and a live birth may be expected.
